# Myasthenia gravis following the initiation of statin therapy: A multinational self‐controlled case series study

**DOI:** 10.1111/joim.70072

**Published:** 2026-02-05

**Authors:** Vincent Ka Chun Yan, Wanchun Xu, Yuta Taniguchi, Kwun Kei Fung, Koon Ho Chan, Gary Kui Kai Lau, Celine Sze Ling Chui, Francisco Tsz Tsun Lai, Xue Li, Masao Iwagami, Rie Masuda, Nanako Tamiya, Sreemanee Raaj Dorajoo, Jing Wei Neo, Esther Wai Yin Chan, Ian Chi Kei Wong, Eric Yuk Fai Wan

**Affiliations:** ^1^ Centre for Safe Medication Practice and Research, Department of Pharmacology and Pharmacy, Li Ka Shing Faculty of Medicine The University of Hong Kong Hong Kong Hong Kong Special Administrative Region, China; ^2^ Department of Family Medicine and Primary Care, School of Clinical Medicine, Li Ka Shing Faculty of Medicine The University of Hong Kong Hong Kong Hong Kong Special Administrative Region, China; ^3^ Department of Health Services Research, Institute of Medicine University of Tsukuba Tsukuba Japan; ^4^ Department of Medicine School of Clinical Medicine Li Ka Shing Faculty of Medicine The University of Hong Kong Hong Kong Hong Kong Special Administrative Region, China; ^5^ School of Nursing, Li Ka Shing Faculty of Medicine The University of Hong Kong Hong Kong Hong Kong Special Administrative Region, China; ^6^ Advanced Data Analytics for Medical Science Limited Hong Kong Hong Kong Special Administrative Region, China; ^7^ Division of Nursing, Higashigaoka Faculty of Nursing Tokyo Healthcare University Shinagawa City Japan; ^8^ Health Sciences Authority Singapore Singapore; ^9^ Department of Pharmacy, Shenzhen Institute of Research and Innovation The University of Hong Kong Shenzhen China; ^10^ Aston Pharmacy School Aston University Birmingham UK; ^11^ School of Pharmacy Medical Sciences Division Macau University of Science and Technology Taipa Macau China; ^12^ The Institute of Cardiovascular Science and Medicine, Li Ka Shing Faculty of Medicine The University of Hong Kong Hong Kong Hong Kong Special Administrative Region, China; ^13^ Comprehensive Primary Healthcare Collaboratory Li Ka Shing Faculty of Medicine The University of Hong Kong Hong Kong Hong Kong Special Administrative Region, China

**Keywords:** myasthenia gravis, self‐controlled case series study, statins

## Abstract

**Background:**

Evidence regarding the risk of new‐onset myasthenia gravis (MG) following statin therapy initiation is limited.

**Objectives:**

To investigate this potential adverse effect using multinational real‐world population‐based data.

**Methods:**

A self‐controlled case series (SCCS) study was conducted using electronic medical records and claims databases from Hong Kong, the United Kingdom (UK) and Japan. Individuals aged ≥18 years with first diagnosis of MG and initiated statins were included. Conditional Poisson regression compared the risk of MG in different risk periods (up to 2 years after initiation) with non‐exposure period, adjusted for age. Pooled results based on meta‐analysis across all study sites were reported.

**Results:**

In total, 2267 MG cases were analysed. Combined across all study sites, a significantly increased risk of incident MG was observed during the first year after statin initiation compared to non‐exposure period, with a higher risk from Days 0–179 (pooled incidence rate ratio [IRR] [95% CI]: 2.662 [1.276–5.553]) than Days 180–364 (1.407 [1.014–1.954]). No increased risk of MG was observed more than 1 year after statin initiation (1.011 [0.848–1.206]). Moreover, the magnitude of MG risk elevation within the first 180 days after statin initiation was more pronounced with higher intensity statin regimens.

**Conclusion:**

In this multinational SCCS study, statin initiation may be associated with increased risk of new‐onset MG during the first 6–12 months, with greater magnitude of risk elevation for higher intensity statin therapy. Consideration of the possibility of new‐onset MG may be advisable within first 6–12 months after initiating statins, especially for medium‐to‐high‐intensity statin therapy.

## Graphical abstract

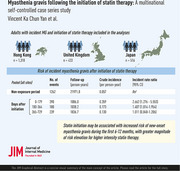

**Abbreviations**
ACC/AHA: American College of Cardiology/American Heart Association
CDARS: Clinical Data Analysis and Reporting System
CIs: confidence intervals
HKSAR: Hong Kong Special Administrative Region
HMGCR: hydroxy‐3‐methylglutaryl‐CoA reductase
IMNM: immune‐mediated necrotizing myopathy
IMRD: IQVIA Medical Research Data
IRRs: incidence rate ratios
MG: myasthenia gravis
SCCS: self‐controlled case series
THIN: The Health Improvement Network
UK: United Kingdom

## Introduction

Statins are the most widely used class of cholesterol‐lowering drug and have long been utilized for primary and secondary prevention of cardiovascular events [1, 2, 3, 4]. However, the potential association between statin therapy and the adverse event of myasthenia gravis (MG) has recently been brought into the attention of regulatory authorities in Europe, the United Kingdom (UK), Hong Kong, Australia and Japan [5, 6, 7, 8, 9]. MG is a rare chronic neuromuscular disorder, with a spectrum of symptoms ranging from purely ocular to severe weakness of respiratory muscles [10], which can be potentially fatal [11]. Although MG is a rare disease, it may lead to a severe decrease in quality of life, as patients may suffer from muscle weakness, double vision, impaired speech and other neuromuscular symptoms [12]. The exact mechanism behind the association of statins and MG remains unclear. Several hypotheses from the literature include increased synthesis of autoantibodies against acetylcholine receptors [2], depletion of the cholesterol involved in synaptic transmission in neuromuscular junctions [10] and direct myotoxicity of statins [2].

A number of case reports and a disproportionality analysis of voluntary adverse drug reaction reports suggested a possible link between statins and MG onset [13, 14, 15, 16, 17, 18, 19], whereas another case‐control study suggested no association between statin use and MG onset [20]. Our previous cohort study based on the Hong Kong population found that statin use was associated with an increased risk of MG onset [21]. However, the majority of Hong Kong's population is Chinese, and thus these findings may be limited in their generalizability to other ethnicities. Furthermore, adverse events could be dose‐dependent, but the previous study had a limited sample size to further evaluate the association between different statin intensities and MG. Therefore, there is a need for further studies with diverse ethnicities and larger sample sizes to provide robust real‐world evidence to inform clinical practice.

In this study, we aim to use a multinational real‐world population‐based electronic health record from three countries (Hong Kong, the UK and Japan) to investigate the risk of new MG onset after initiation of statin therapy, using a self‐controlled case series (SCCS) study design for within‐individual comparison in order to produce more robust results across diverse ethnic populations.

## Methods

### Data sources

This was a multinational study using data from Hong Kong, the UK and Japan.

The Clinical Data Analysis and Reporting System (CDARS) is a territory‐wide electronic medical records database from the Clinical Management System of the Hospital Authority of Hong Kong. The Hospital Authority is the statutory administrative organization managing the public healthcare sector in Hong Kong. The Hospital Authority is funded by the Hong Kong Special Administrative Region (HKSAR) Government and provides subsidized clinical services (outpatient, hospitalization and emergency department) to over 7.4 million Hong Kong residents. For hospitalization services, HA covers approximately 80% of all hospital admissions in Hong Kong [22]. The Clinical Management System provides real‐time information to support day‐to‐day patient care operations across all public hospitals and clinics. CDARS contains data covering all aspects of patient care, including patients’ demographics, diagnoses, procedures, prescriptions, laboratory tests, inpatient admissions, outpatient and emergency department attendances. CDARS is an internationally recognized database with territory‐wide population coverage and comprehensive coverage of various aspects of patient care and has been used in numerous healthcare big data research studies [23, 24, 25, 26, 27, 28]. Previous studies demonstrated a high degree of coding accuracy for a variety of outcomes [24, 27, 29].

The IQVIA Medical Research Data (IMRD)‐UK database, which incorporates data from The Health Improvement Network (THIN), a Cegedim Database [30], is a nationwide database of primary care records in the UK that includes around 6% of the total UK population. This work used de‐identified data provided by patients as a part of their routine primary care from within this database. Previous studies have demonstrated the validity of the database for pharmacoepidemiologic studies and generalizability to the UK population [31, 32]. The IMRD‐UK database includes data that are recorded in primary care settings on demographic information, lifestyle information, medical diagnosis and procedures (recorded in Read codes), laboratory test values and prescribing information.

The JMDC Claims Database, in Japan, is a claims database from health insurance associations for large‐ and medium‐sized company employees and their dependent family members (aged <75 years), with approximately 16 million individuals included [33]. Previous studies have demonstrated the validity of the database for pharmacoepidemiologic studies [34], and this database has been used in various healthcare big data research [35, 36].

### Study design

This study used an SCCS design to evaluate the risk of MG after statin initiation. The SCCS study design is a within‐individual comparison based on a case‐only approach [37]. In an SCCS design, participants serve as their own control, and relative risk is estimated based on within‐person rather than between‐person comparisons by comparing the rate of outcome events between different risk windows and the baseline period of the same individual. The major advantage of SCCS over other between‐individual comparison study designs (such as cohort study) is that it eliminates both measured and unmeasured time‐invariant confounders that vary between individuals, such as family history, genetic factors, socioeconomic status and underlying disease severity [37]. Furthermore, time‐varying factors, such as age and season, could be adjusted for. The SCCS study design has been applied in numerous post‐marketing pharmacovigilance studies evaluating rare adverse effects of medications and vaccines [29, 38, 39, 40, 41, 42].

There are a few major assumptions of the SCCS design that need to be met in this study. First, events should arise independently within individuals if recurrent, or events are uncommon if non‐recurrent. As we could not rule out the possibility that a first MG episode increases the chance of a second, nor reliably differentiate a new MG episode versus follow‐up care of an existing MG episode, only the first‐ever MG event was considered in this study, and cases with a history of MG before the start of the observation period were excluded to meet this assumption. Second, events should not influence the probability of subsequent exposure. During the study period, MG had not been considered a contraindication for prescribing statins according to international guidelines. Therefore, the presence of pre‐existing MG should not influence the likelihood of initiating statin therapy. Nevertheless, a pre‐exposure period of 180 days was added in the main analyses to account for the possibility of event‐dependent exposure. Third, events shall not censor the observation period. The fatality rate of MG is relatively low. Sensitivity analyses were also conducted by excluding patients who died during the observation period to mitigate this possibility.

### Study population

As this is an SCCS analysis, only exposed cases (i.e., patients with MG and statin exposure at any time during the study period) need to be analysed. Therefore, individuals with their first diagnosis of MG (identified using Read codes F380.00, F380100 and F380z00 for the UK, ICD‐9 code 358.0* for HK and ICD‐10 code G700* for Japan) and initiated any statin (first‐ever statin prescription) during the study period (HK: 1 January 2004–30 November 2023, UK: 1 January 2000–30 November 2021, Japan: 1 January 2005–31 March 2023) were included, and individuals aged less than 18 years or who had a history of MG before the start of the study period were excluded. Additionally, for UK data, only individuals with at least 1 year of up‐to‐standard data record in the database were included. Thus, the look‐back period to exclude pre‐existing MG began from the start of data availability in the database till the first occurrence of MG during the study period, which was at least 12 months for all individuals.

### Exposure and outcomes

The exposure of interest in this study was statin use, defined as the prescription of any statin. The primary outcome was incident diagnosis of MG. The relevant case definitions for MG in different databases were detailed in Table S1. In the main analyses, only one diagnosis code was required to be categorized as an MG event. A sensitivity analysis was carried out as well, where a minimum of two diagnosis codes at different time points was required to be categorized as an MG event.

### Definitions of observation period and risk periods

**Fig. 1:**
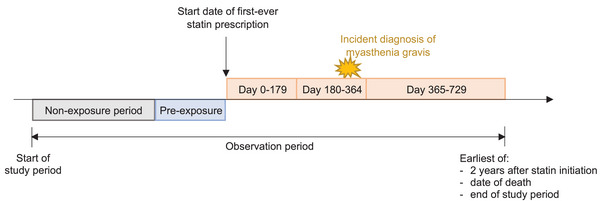
Study design.

Individuals were followed up from the start of the study period (or 1 year after the patient's registration to general practice for UK data) till the earliest of (i) 2 years after statin initiation, (ii) date of death and (iii) end of study period (or date of transfer out from general practice for UK data). Person‐time during the observation period was divided into discrete risk windows based on the number of days after statin initiation, as follows: Days 0–179, Days 180–364 and Days 365–729 after the start date of the first‐ever statin prescription. A pre‐exposure period of 180 days before statin initiation was added to account for the possibility of event‐dependent exposure, with sensitivity analyses varying the duration of the pre‐exposure period. Periods of follow‐up that are not risk periods were classified as non‐exposure (reference) periods (Fig.  1).

### Statistical analysis

The SCCS model was adjusted for age (categorized in 5‐year bands from 30 to 80 years), which served as the primary time‐varying confounder for statin exposure and MG. As SCCS is a within‐individual comparison, clusters of dependent data are defined at the individual patient level. Crude incidence rates for outcomes in each risk window were reported. It should be noted that these crude incidence rates should only be interpreted as relative to incidence rates in other risk periods or the non‐exposure period and do not represent the absolute incidence rate in the general population, because this is a case‐only study without information on statin non‐users or people without MG outcomes. Conditional Poisson regression was used to compare the incidence rates in each risk window with reference periods. Incidence rate ratios (IRRs) and 95% confidence intervals (CIs) for each risk window were estimated. Pooled IRR with 95% CI was calculated separately for each risk period based on a meta‐analysis across all study sites with random effects model, and the heterogeneity of study estimates was assessed using *I* ^2^. Analyses stratified by sex, statin intensity based on American College of Cardiology/American Heart Association (ACC/AHA) guideline [43] (Table S4), and individual statin drugs were conducted. Sensitivity analyses were conducted by (i) ending the observation period at 30 days after discontinuation of statins (if earlier); (ii) excluding patients who died during the observation period; (iii) treating Day 0 as a separate risk period; (iv) excluding events that happen on the same day of statin initiation; and (v) varying the duration of the pre‐exposure risk period as 90 or 365 days. All analyses were conducted in R using the ‘SCCS’ and ‘metafor’ packages. A *p* value of less than 0.05 was considered statistically significant.

## Results

**Fig. 2:**
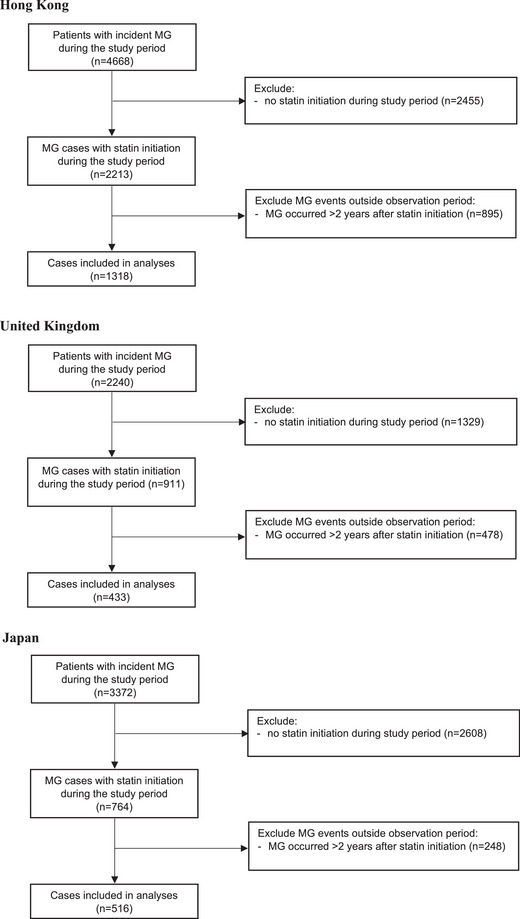
Flowchart for selection of self‐controlled case series (SCCS) cases. MG, myasthenia gravis.

A total of 2267 MG cases across three study sites were included in the analyses (Fig. 2). The mean (SD) age at the beginning of the observation period was between 50.75 (12.50) and 59.45 (11.50) across the three study sites, and 48.4%–63.7% were male. The mean (SD) duration of follow‐up was between 5.33 (3.80) and 14.97 (4.55) years, and 0.4%–6.9% died during the observation period. The mean (SD) duration of statin treatment was between 2.54 (2.31) and 4.07 (3.94) years (Table 1).

**Table 1:** Characteristics of cases included in the self‐controlled case series (SCCS).

	Hong Kong	United Kingdom	Japan
Number of cases	1318	433	516
Age, years—mean (SD)	50.75 (12.50)	59.45 (11.50)	52.15 (10.24)
Sex, male—no. (%)	638 (48.4)	276 (63.7)	289 (56.0)
Duration of follow‐up, years—mean (SD)	14.97 (4.55)	10.40 (5.11)	5.33 (3.80)
Duration of statin treatment, years—mean (SD)	4.07 (3.94)	3.11 (3.62)	2.54 (2.31)
Died during observation period—no. (%)	53 (4.0)	30 (6.9)	2 (0.4)
Statin drug—no. (%)			
Atorvastatin	356 (27.0)	132 (30.5)	139 (26.9)
Cerivastatin	0 (0.0)	1 (0.2)	0 (0.0)
Fluvastatin	3 (0.2)	3 (0.7)	1 (0.2)
Pitavastatin	0 (0.0)	0 (0.0)	93 (18.0)
Pravastatin	0 (0.0)	12 (2.8)	47 (9.1)
Rosuvastatin	48 (3.6)	8 (1.8)	229 (44.4)
Simvastatin	911 (69.1)	277 (64.0)	7 (1.4)
Statin intensity—no. (%)			
Low	694 (52.7)	49 (11.3)	314 (60.9)
Medium	592 (44.9)	343 (79.4)	202 (39.1)
High	32 (2.4)	40 (9.3)	0 (0.0)
Unknown	0 (0.0)	1 (0.2)	0 (0.0)

Combined across all study sites, a significantly increased risk of incident MG was observed during the first year after statin initiation compared to the non‐exposure period (Table 2), with a higher risk from Days 0 to 179 (pooled IRR [95% CI]: 2.662 [1.276–5.553]) than Days 180–364 (pooled IRR [95% CI]: 1.407 [1.014–1.954]). No increased risk of MG was observed 1 year after statin initiation compared to the non‐exposure period (pooled IRR [95% CI]: 1.011 [0.848–1.206]). For individual study sites, a significantly increased risk of incident MG was observed during the first year after statin initiation in Hong Kong (IRR [95% CI] for Days 0–179: 4.667 [3.911–5.569], Days 180–365: 1.573 [1.215–2.036]) and the UK (IRR [95% CI] for Days 0–179: 3.023 [2.212–4.131], Days 180–365: 1.759 [1.209–2.560]). Notably, the IRRs and CIs for the increased risk observed in Hong Kong and the UK during the first 180 days did not overlap with those for Japan, where only a non‐significant trend towards increased risk of MG was seen (IRR [95% CI]: 1.302 [0.943–1.796]) (Table 2).

**Table 2:** Risk of incident myasthenia gravis after initiation of statin therapy.

	No. of events	Follow‐up (person‐years)	Crude incidence (per person‐year)^a^	Incident rate ratio (95% CI)
**Pooled (all sites)** ^b^				
0–179 days after initiation	390	1086.0	0.359	2.662 (1.276–5.553)
180–364 days after initiation	180	1038.2	0.173	1.407 (1.014–1.954)
365–729 days after initiation	239	1836.7	0.130	1.011 (0.848–1.206)
Non‐exposure period	1262	21,971.8	0.057	(Ref.)
**Hong Kong**				
0–179 days after initiation	228	629.6	0.362	4.667 (3.911–5.569)
180–364 days after initiation	76	602.3	0.126	1.573 (1.215–2.036)
365–729 days after initiation	99	1090.1	0.091	1.065 (0.838–1.355)
Non‐exposure period	834	16,767.5	0.050	(Ref.)
**United Kingdom**				
0–179 days after initiation	73	211.1	0.346	3.023 (2.212–4.131)
180–364 days after initiation	42	205.4	0.204	1.759 (1.209–2.560)
365–729 days after initiation	46	370.4	0.124	1.013 (0.692–1.483)
Non‐exposure period	228	3506.2	0.065	(Ref.)
**Japan**				
0–179 days after initiation	89	245.2	0.363	1.302 (0.943–1.796)
180–364 days after initiation	62	230.4	0.269	0.990 (0.692–1.418)
365–729 days after initiation	94	376.2	0.250	0.903 (0.635–1.284)
Non‐exposure period	200	1698.1	0.118	(Ref.)

^a^ The reported crude incidence rates should only be interpreted as relative to incidence rates in other risk periods or the non‐exposure period and do not represent the absolute incidence rate in the general population, because this is a case‐only study without information on statin non‐users or people without MG outcomes.

^b^ *I* ^2^ for pooled incident rate ratio = 96% (Days 0–179), 67% (Days 180–364), 0% (Days 365–729). Additionally, there were 196 events during the pre‐risk period (Hong Kong: 81, UK: 44, Japan: 71), which was included in the analyses but not displayed in this summary table. Concordance = 0.71.

The magnitude of risk elevation of MG within the first 180 days after statin initiation was more pronounced with higher intensity statin regimens (pooled IRR [95% CI] for high‐intensity: 4.826 [2.175–10.710], medium‐intensity: 2.940 [1.434–6.028], low‐intensity: 2.179 [0.969–4.898]) (Table 3). Similarly, a significant increase in risk of MG was observed during Days 180–365 after initiation of medium‐intensity statin therapy (pooled IRR [95% CI]: 1.529 [1.182–1.978]), but the increase in risk was not significant for low‐intensity statin therapy (pooled IRR [95% CI]: 1.340 [0.882–2.036]). The increase in risk of MG during the first 180 days after statin initiation was consistently observed across analyses stratified by sex (pooled IRR [95% CI] for male: 2.619 [1.228–5.584], female: 2.695 [1.301–5.580]) and individual statins (pooled IRR [95% CI] for simvastatin: 4.105 [3.380–4.985], atorvastatin: 2.469 [0.889–6.857], rosuvastatin: 3.287 [0.914–11.821]) (Table 3). Findings consistent with the main analyses were observed across all sensitivity analyses (Tables S2 and S3), including those with different pre‐risk periods, which suggested that our main findings were robust to different definitions of incident MG and possible diagnostic delay.

**Table 3:** Subgroup analyses—pooled results across all sites.

	No. of events	Follow‐up (person‐years)	Crude incidence (per person‐year)^a^	Incident rate ratio (95% CI)
**Sex**				
*Male*				
0–179 days after initiation	209	580.8	0.360	2.619 (1.228–5.584)
180–364 days after initiation	112	559.0	0.200	1.548 (1.101–2.175)
365–729 days after initiation	143	986.9	0.145	1.058 (0.763–1.467)
Non‐exposure period	626	11,332.4	0.055	(Ref.)
*Female*				
0–179 days after initiation	181	505.1	0.358	2.695 (1.301–5.580)
180–364 days after initiation	68	479.2	0.142	1.218 (0.914–1.623)
365–729 days after initiation	96	849.9	0.113	0.877 (0.665–1.155)
Non‐exposure period	636	10,639.3	0.060	(Ref.)
**Statin intensity**				
*Low‐intensity*				
0–179 days after initiation	171	508.8	0.336	2.179 (0.969–4.898)
180–364 days after initiation	87	494.2	0.176	1.340 (0.882–2.036)
365–729 days after initiation	124	895.3	0.139	0.971 (0.614–1.537)
Non‐exposure period	582	9225.5	0.063	(Ref.)
*Medium‐intensity*				
0–179 days after initiation	207	541.5	0.382	2.940 (1.434–6.028)
180–364 days after initiation	90	510.7	0.176	1.529 (1.182–1.978)
365–729 days after initiation	112	883.6	0.127	0.972 (0.693–1.364)
Non‐exposure period	634	11,766.8	0.054	(Ref.)
*High‐intensity*				
0–179 days after initiation	11	35.1	0.313	4.826 (2.175–10.710)
180–364 days after initiation	3	32.8	0.091	1.274 (0.356–4.561)
365–729 days after initiation	3	24.6	0.122	2.987 (0.610–14.624)
Non‐exposure period	46	979.5	0.047	(Ref.)
**Drug**				
*Simvastatin*				
0–179 days after initiation	204	577.3	0.353	4.105 (3.380–4.985)
180–364 days after initiation	87	573.9	0.152	1.687 (1.312–2.168)
365–729 days after initiation	115	1078.1	0.107	1.129 (0.891–1.430)
Non‐exposure period	710	12,399.0	0.057	(Ref.)
*Atorvastatin*				
0–179 days after initiation	105	294.9	0.356	2.469 (0.889–6.857)
180–364 days after initiation	44	265.7	0.166	1.221 (0.802–1.859)
365–729 days after initiation	58	427.9	0.136	0.897 (0.620–1.298)
Non‐exposure period	369	7520.7	0.049	(Ref.)
*Rosuvastatin*				
0–179 days after initiation	51	133.6	0.382	3.287 (0.914–11.821)
180–364 days after initiation	28	120.3	0.233	1.206 (0.540–2.692)
365–729 days after initiation	39	166.3	0.235	0.971 (0.364–2.591)
Non‐exposure period	123	1486.7	0.082	(Ref.)

^a^The reported crude incidence rates should only be interpreted as relative to incidence rates in other risk periods or the non‐exposure period and do not represent the absolute incidence rate in the general population, because this is a case‐only study without information on statin non‐users or people without MG outcomes.

## Discussion

In this multinational SCCS study, we observed an approximately 2.7‐fold and 1.4‐fold increased risk of new‐onset MG within the first 6 months and 6–12 months after initiating statin therapy, respectively. Furthermore, we observed a greater magnitude of risk elevation with higher intensity statin therapy. However, our study did not find any increased risk of MG beyond 12 months after initiating statin therapy. These findings suggest the importance of considering the possibility of new‐onset MG for a duration of 12 months after initiating statin therapy, especially among individuals receiving high‐intensity statin regimens who present with persistent muscular symptoms that do not resolve after discontinuation of statin therapy for suspected statin‐induced myopathy. Notably, the increased risk observed in Hong Kong and the UK was not statistically significant in Japan, which may be due to heterogeneity in healthcare systems, population age structure, database coverage and case ascertainment across different countries. Nevertheless, the absolute incidence rate of MG in the general population is known to be low. The average annual incidence of MG in Hong Kong, the UK and Japan was 0.4, 2.46 and 0.87 per 100,000 person‐years, respectively, and the prevalence was 6.3, 33.7 and 23.1 per 100,000 population [44, 45, 46, 47]. This variation likely reflects, at least in part, differences in the timing (1992, 2021 and 2022 for Hong Kong, the UK and Japan, respectively) and the quality and nature of the underlying data as well as the methods used for case ascertainment. Over time, improvements in disease awareness, diagnostic criteria, population demographics, environmental factors and healthcare access may have contributed to higher detection rates in these later studies [47]. Unfortunately, we were unable to identify more recent epidemiological studies of MG in Hong Kong, limiting our ability to directly compare these rates with those from the UK and Japan. Considering the rarity of MG as a potential adverse event or as a condition that may be unmasked or accelerated by statin therapy, the potential benefits of statin therapy are expected to outweigh the associated risk.

Our findings contrast with those of a previous case‐control study involving 190 subjects, which did not find a significant association between statin use and the onset of MG (OR 1.70, 95% CI 0.60–4.87) [20]. This discrepancy may be attributed to the limited statistical power of the earlier study to detect an increased risk of MG onset. In comparison, our multinational population‐based SCCS study included a larger sample size, enabling us to achieve sufficient statistical power to detect the risk and provide more robust results across different populations, while applying an intra‐individual comparison to minimize unmeasured confounders compared to a case‐control study design. Indeed, a prior case‐series study reported that 6 out of 54 (11%) statin users experienced worsening MG symptoms or MG relapse within 4 months of statin initiation [48]. Additionally, a study using disproportionality analysis also indicated a signal for an association between MG and statin use (ROR 2.66, 95% CI 2.28–3.10) [19]. Moreover, our previous cohort study demonstrated that the increased risks of MG onset were observed within the first 6 months following statin initiation, which was consistent with the findings in the current study based on a larger number of events (∼1300 compared to ∼200 in the previous study) [21]. Meanwhile, on top of literature, our multinational SCCS study included a larger sample size by broadening the study period and utilizing datasets across different populations. By employing an advanced scientific approach with multinational databases, our study provided real‐world evidence to confirm the signal of an increased risk of MG after statin initiation. Moreover, we found that the magnitude of MG risk elevation within the first 180 days after initiating statin therapy was more pronounced with higher intensity statin regimens. Nevertheless, whether combinations of low‐intensity statins with other lipid‐lowering agents such as ezetimibe or bempedoic acid may limit the potential onset effects of MG warrants further study.

Statin therapy is known to induce immune‐mediated necrotizing myopathy (IMNM) via induction of autoantibodies targeting 3‐hydroxy‐3‐methylglutaryl‐CoA reductase (HMGCR) enzyme [49]. Yet, the exact mechanism behind the association of statins and MG is unclear, although there are several hypotheses from different literature. Some suggest that statins may induce production of cytokines, leading to increased synthesis of autoantibodies against acetylcholine receptors [2]. Statins may deplete coenzyme Q10 and lead to dysfunction of mitochondria [2]. Th2 cells may also play a role, as they increase the activity of antibody when upregulated by statins [1, 18]. Other studies found that statin withdrawal leads to the disappearance of autoantibodies against acetylcholine receptors in some cases [3, 19]. Furthermore, statins may deplete the cholesterol involved in the process of the synaptic transmission in neuromuscular junctions [10]. The direct myotoxicity of statins may also contribute to muscle weakness as well [2]. Moreover, the underlying mechanisms for a potential dose‐response relationship between statin intensity and risk of MG as shown in our study had not been discussed in the current literature. Among the very few MG case reports which reported the dosage of statin used, all of them used a moderate‐ or high‐intensity statin [16, 17, 18]. Further research is warranted to better understand the potential mechanisms behind the association between statin initiation and new‐onset MG.

This study is the first multinational study investigating the risks of new‐onset MG after statin initiation. The use of a within‐individual study design eliminates potential bias due to time‐invariant confounders, which was a common limitation in previous research based on inter‐individual comparisons. The increase in risk of new‐onset MG after statin initiation was consistently observed across multiple study sites, which included patients of different ethnicities, suggesting the generalizability of our findings. Furthermore, our results suggest a dose‐dependent relationship of the risk of new‐onset MG, which further strengthened our conclusions. Nevertheless, there were a few limitations of this study. First, the current findings suggest an association but could not confirm a causal relationship between statin use and risk of MG. As with all observational studies using electronic medical records, we can only assess temporal associations between statin initiation and subsequent diagnosis of MG. We cannot determine whether statins directly cause MG or whether statin‐associated muscle symptoms prompt further clinical investigations, leading to increased detection or diagnosis of pre‐existing but previously unrecognized MG (diagnostic bias or unmasking effect) [50]. Further large epidemiological case‐control studies are warranted to confirm the results. Second, the possibility of time‐varying confounding (e.g., due to COVID‐19, other co‐medications or comorbidity burden) could not be ruled out. Nevertheless, age has been adjusted for in our statistical models. Third, the sample size for certain subgroups was limited, and there was a high heterogeneity of estimates across study sites. Fourth, due to the nature of electronic medical records databases used in this study, information was not available regarding the type of MG (e.g., ocular vs. generalized), the presence and subtype of antibodies (e.g., anti‐AChR and anti‐MuSK antibodies) or the severity and clinical course of MG cases. As a result, there is a possibility that some patients with new‐onset muscular symptoms may have been initially misclassified as MG before full diagnostic confirmation. There may also be delays from symptom onset to actual diagnosis of MG, although the long risk windows in this study shall ensure all relevant events were captured. Furthermore, there is risk of misclassification based on neuromuscular symptoms that may resemble MG but turn out to be a different diagnosis. Nevertheless, we had conducted sensitivity analyses where at least 2 diagnosis records at different time points were required to define MG, and the results were consistent with the main analyses. Yet the risk of such misclassification cannot be ruled out. Fifth, misdiagnosis of MG is possible, particularly for the JMDC claims database, where investigations (e.g., electromyography) for differential diagnosis of MG following muscle symptoms after starting statins may be recorded in the claims database for reimbursement purposes. On the other hand, Hong Kong (CDARS) and the UK (THIN) databases are electronic medical records, where diagnosis coding is performed by clinicians for routine patient care after thorough examination and confirmation of the diagnosis, and hence the possibility of misdiagnosis of MG for Hong Kong and the UK is likely minimal. Future studies with more granular clinical data are warranted to improve the fidelity of MG diagnosis. In addition, as an insurer‐based claims database, the JMDC database is subject to selection bias towards younger and employed individuals, limiting the generalizability of these findings to the wider Japanese population. Lastly, the patient adherence data are unavailable in the current databases. Nevertheless, as statins are considered one of the first‐line therapies for primary and secondary prevention of major adverse cardiovascular events, relatively high adherence to statin treatment was generally observed in Hong Kong, Japan, and the UK. Moreover, the results from sensitivity analyses, which considered statin prescriptions over the observation period by ending it 30 days after statin discontinuation, were consistent with the main analyses.

## Conclusion

In this multinational SCCS study, statin initiation may be associated with a significantly increased risk of incident MG during the first 6–12 months, with a greater magnitude of risk elevation for higher intensity of statin therapy. Considering the rarity of MG as an adverse event, the potential benefits of statin therapy are expected to outweigh the associated risk. Consideration of the possibility of new‐onset MG may be advisable within the first 6–12 months after initiation of statins, especially for medium‐to‐high‐intensity statin therapy.

## Author contributions

Concept and design: Eric Yuk Fai Wan, Vincent Ka Chun Yan, Wanchun Xu, Yuta Taniguchi, Masao Iwagami, Ian Chi Kei Wong and Esther Wai Yin Chan. Acquisition, analysis or interpretation of data: Vincent Ka Chun Yan, Eric Yuk Fai Wan, Yuta Taniguchi, Masao Iwagami, Koon Ho Chan, Gary Kui Kai Lau, Jing Wei Neo, Sreemanee Raaj Dorajoo, Ian Chi Kei Wong and Esther Wai Yin Chan. Drafting of the manuscript: Vincent Ka Chun Yan, Eric Yuk Fai Wan, Kwun Kei Fung, Yuta Taniguchi and Masao Iwagami. Critical revision of the manuscript for important intellectual content: All authors. Statistical analysis: Vincent Ka Chun Yan and Yuta Taniguchi. Administrative, technical or material support: Eric Yuk Fai Wan, Masao Iwagami, Ian Chi Kei Wong and Esther Wai Yin Chan. Supervision: Eric Yuk Fai Wan, Masao Iwagami, Ian Chi Kei Wong and Esther Wai Yin Chan. Eric Yuk Fai Wan had full access to all the data in the study and takes responsibility for the integrity of the data and the accuracy of the data analysis.

## Conflict of interest statement

Eric Yuk Fai Wan has received research grants from the Health Bureau, the Hong Kong Research Grants Council, the Narcotics Division, the Security Bureau, the Social Welfare Department, the Labour and Welfare Bureau of the Government of the Hong Kong SAR and the National Natural Science Foundation of China; serves as a member of the Core Team for Expert Group on Drug Registration of the Pharmacy and Poisons Board and is the director of Advance Data Analytics for Medical Science (ADAMS) Limited (HK). These are outside the submitted work.

Ian Chi Kei Wong received research grants from Amgen, Janssen, GSK, Novartis, Pfizer, Bayer and Bristol‐Myers Squibb and Takeda, the Institute for Health Research in England, the European Commission, the National Health and Medical Research Council in Australia, the European Union's Seventh Framework Programme for research, technological development, the Research Grants Council Hong Kong and the Health and Medical Research Fund Hong Kong; consulting fees from IQVIA and the World Health Organization; payment for expert testimony for Appeal Court in Hong Kong; serves on advisory committees for the Member of Pharmacy and Poisons Board; and is a member of the Expert Committee on Clinical Events Assessment, following COVID‐19 immunization; is a member of the Advisory Panel on COVID‐19 Vaccines of the Hong Kong Government; is the non‐executive director of Jacobson Pharma Corp. Ltd. in Hong Kong; and is the founder and director of Therakind Limited (UK), Advance Data Analytics for Medical Science (ADAMS) Limited (HK) and OCUS Innovation Limited (HK, Ireland and UK).

Esther Wai Yin Chan reports grants from the Hong Kong Research Grants Council of the Government of the Hong Kong SAR, the Research Fund Secretariat of the Food and Health Bureau, the National Natural Science Fund of China, Bayer, Bristol‐Myers Squibb, Pfizer, Janssen, Amgen, Takeda, RGA Reinsurance Company, AstraZeneca, the Narcotics Division of the Security Bureau of the Hong Kong Special Administrative Region, the Innovation and Technology Commission of the Government of the Hong Kong Special Administrative Region, Novartis, the National Health and Medical Research Council Australia, and honorarium from the Hospital Authority, outside the submitted work.

Celine Sze Ling Chui has received grants from the Food and Health Bureau of the Hong Kong Government, the Hong Kong Research Grant Council, the Hong Kong Innovation and Technology Commission, Pfizer, IQVIA, MSD and Amgen; is the CEO of ADAMS Limited (HK); and has received personal fees from MSD, and PrimeVigilance outside the submitted work.

Xue Li received research grants or contracts from the Health and Medical Research Fund (HMRF Main Scheme, HMRF Fellowship Scheme, and Hong Kong Special Administrative Region) and from the Research Grants Council Early Career Scheme (HKSAR); is also the former non‐executive director of ADAMS Hong Kong; received commission grants from Hospital Authority of Hong Kong, internal funding from the University of Hong Kong, and research or education grants from Pfizer, Janssen and Bristol Myers Squibb (BMS), and Novartis; received consultancy fees from Merck Sharp & Dohme, Pfizer, Novartis, Open Health and the Office of Health Economics; and received honoraria for associate editorship from Nature Springer.

No other disclosures were reported.

## Funding information

This work was supported by Health and Medical Research Fund. ICKW and FTTL are partially supported by the Laboratory of Data Discovery for Health (D24H) funded by the AIR@InnoHK administered by Innovation and Technology Commission. The funders had no role in the design and conduct of the study; collection, management, analysis, and interpretation of the data; preparation, review, or approval of the manuscript; and decision to submit the manuscript for publication.

## Ethics statement

This study was conducted in accordance with the principles outlined in the Declaration of Helsinki. This study was approved by the Institutional Review Board of the University of Hong Kong/Hospital Authority Hong Kong West Cluster (ref: UW 19‐640), the IMRD Scientific Review Committee (ref: 23SRC033) and the Ethics Committee of the University of Tsukuba (ref: 1965).

## Consent

The data used in this study were fully anonymized before we accessed them, therefore, informed consent was not required according to the guidelines of the institutional review boards and ethics committees.

## Supporting information




**Table S1**: Case definitions of myasthenia gravis.
**Table S2**: Subgroup and sensitivity analyses results of each individual site Hong Kong.
**Table S3**: Sensitivity analyses pooled results across all sites.
**Table S4**: Definitions for statin intensity.

## Data Availability

The electronic medical records used in the current are provided by the Hospital Authority of Hong Kong, IQVIA and JMDC. The data can be accessed upon request to the Hospital Authority of Hong Kong, IQVIA and JMDC.

## References

[1] Attardo S , Musumeci O , Velardo D , Toscano A . Statins neuromuscular adverse effects. Int J Mol Sci. 2022;23(15):8364. 35955495 10.3390/ijms23158364 PMC9369175

[2] Andronie‐Cioară FL , Jurcău A , Jurcău MC , Nistor‐Cseppentö DC , Simion A . Cholesterol management in neurology: time for revised strategies? J Pers Med. 2022;12(12):1981. 36556202 10.3390/jpm12121981 PMC9784893

[3] Loh WJ , Watts GF . The management of hypercholesterolemia in patients with neuromuscular disorder. Curr Atheroscler Rep. 2023;25(2):43–53. 36609642 10.1007/s11883-022-01077-9

[4] Guadamuz JS , Shooshtari A , Qato DM . Global, regional and national trends in statin utilisation in high‐income and low/middle‐income countries, 2015–2020. BMJ Open. 2022;12(9):e061350. 10.1136/bmjopen-2022-061350 PMC9462115 36691204

[5] European Medicine Agency . PRAC recommendations on signals: adopted at the 9–12 January 2023 PRAC meeting . Amsterdam: European Medicine Agency; 2023.

[6] Medicines and Healthcare Products Regulatory Agency . Statins: very infrequent reports of myasthenia gravis . London: Medicines and Healthcare Products Regulatory Agency; 2023. https://www.gov.uk/drug‐safety‐update/statins‐very‐infrequent‐reports‐of‐myasthenia‐gravis#:~:text=before%20taking%20a%20statin%2C%20inform,swallowing%2C%20or%20shortness%20of%20breath.

[7] Drug Information and Pharmacovigilance Division . Statins: very infrequent reports of myasthenia gravis. Hong Kong: Department of Health Drug Office Drug Information and Pharmacovigilance Division; 2023.

[8] Department of Health, Disability and Ageing Therapeutic Goods Administration . Product information safety updates—November 2023 . Canberra: Department of Health, Disability and Ageing Therapeutic Goods Administration; 2023. https://www.tga.gov.au/news/safety‐updates/product‐information‐safety‐updates‐november‐2023.

[9] Pharmaceuticals and Medical Devices Agency . Summary of investigation results: preparations containing HMG‐CoA reductase inhibitor . Tokyo: Pharmaceuticals and Medical Devices Agency; 2023.

[10] Paz ML , Barrantes FJ . Cholesterol in myasthenia gravis. Arch Biochem Biophys. 2021;701:108788. 33548213 10.1016/j.abb.2021.108788

[11] Wendell LC , Levine JM . Myasthenic crisis. Neurohospitalist. 2011;1(1):16–22. 23983833 10.1177/1941875210382918 PMC3726100

[12] National Health Service . Myasthenia gravis . Leeds: National Health Service; 2023. https://www.nhs.uk/conditions/myasthenia‐gravis/.

[13] Keogh MJ , Findlay JM , Leach S , Bowen J . Statin‐associated weakness in myasthenia gravis: a case report. J Med Case Rep. 2010;4:61. 20170525 10.1186/1752-1947-4-61 PMC2834677

[14] Cartwright MS , Jeffery DR , Nuss GR , Donofrio PD . Statin‐associated exacerbation of myasthenia gravis. Neurology. 2004;63(11):2188. 10.1212/01.wnl.0000145708.03876.c3 15596782

[15] Purvin V , Kawasaki A , Smith KH , Kesler A . Statin‐associated myasthenia gravis: report of 4 cases and review of the literature. Medicine (Baltimore). 2006;85(2):82–85. 16609346 10.1097/01.md.0000209337.59874.aa

[16] Khalid R , Ibad A , Thompson PD . Statins and myasthenia gravis. Muscle Nerve. 2016;54(3):509. 10.1002/mus.25155 27105400

[17] Frasson E , Simonetto M , Bertolasi L , Caneve G , Vilotti C , Ruzza G , et al. Statin‐associated necrotizing autoimmune myopathy with concurrent myasthenia gravis. Clin Case Rep. 2021;9(5):e03925. 34026125 10.1002/ccr3.3925 PMC8117815

[18] Gale J , Danesh‐Meyer HV . Statins can induce myasthenia gravis. J Clin Neurosci. 2014;21(2):195–197. 24433954 10.1016/j.jocn.2013.11.009

[19] Gras‐Champel V , Batteux B , Masmoudi K , Liabeuf S . Statin‐induced myasthenia: a disproportionality analysis of the WHO's VigiBase pharmacovigilance database. Muscle Nerve. 2019;60(4):382–386. 31298743 10.1002/mus.26637

[20] Virgo J , Wong S , Rantell K , Plant G . Statins can cause myasthenia gravis: fact or fiction? J Neurol Neurosurg Psychiatry. 2013;84(11):e2.25346970

[21] Xu W , Yan VKC , Zhang Z , Fung KK , Chan KHo , Lau KK , et al. Myasthenia gravis following statin therapy: evidence from target trial emulation and self‐controlled case series study. Nat Commun. 2024;15(1):10317. 39609410 10.1038/s41467-024-54097-1 PMC11604770

[22] HKSAR Government Health Bureau . Hong Kong healthcare systems and healthcare professionals . Hong Kong: HKSAR Government Health Bureau; 2017. https://www.healthbureau.gov.hk/download/press_and_publications/otherinfo/180500_sr/e_ch1.pdf.

[23] Lai EC‐C , Man KKC , Chaiyakunapruk N , Cheng C‐L , Chien H‐C , Chui CSL , et al. Brief report: databases in the Asia‐Pacific region: the potential for a distributed network approach. Epidemiology. 2015;26(6):815–820. 26133022 10.1097/EDE.0000000000000325

[24] Law SWY , Lau WCY , Wong ICK , Lip GYH , Mok MT , Siu C‐W , et al. Sex‐based differences in outcomes of oral anticoagulation in patients with atrial fibrillation. J Am Coll Cardiol. 2018;72(3):271–282. 30012320 10.1016/j.jacc.2018.04.066

[25] Lau WCY , Cheung C‐L , Man KKC , Chan EW , Sing CW , Lip GYH , et al. Association between treatment with Apixaban, Dabigatran, Rivaroxaban, or warfarin and risk for osteoporotic fractures among patients with atrial fibrillation. Ann Intern Med. 2020;173(1):1–9. 32423351 10.7326/M19-3671

[26] Man KKC , Chan EW , Ip P , Coghill D , Simonoff E , Chan PKL , et al. Prenatal antidepressant use and risk of attention‐deficit/hyperactivity disorder in offspring: population based cohort study. BMJ. 2017;357:j2350. 28566274 10.1136/bmj.j2350 PMC5450015

[27] Man KKC , Coghill D , Chan EW , Lau WCY , Hollis C , Liddle E , et al. Association of risk of suicide attempts with methylphenidate treatment. JAMA Psychiatry. 2017;74(10):1048–1055. 28746699 10.1001/jamapsychiatry.2017.2183 PMC5710471

[28] Chai Yi , Luo H , Wong GHY , Tang JYM , Lam T‐C , Wong ICK , et al. Risk of self‐harm after the diagnosis of psychiatric disorders in Hong Kong, 2000–10: a nested case‐control study. Lancet Psych. 2020;7(2):135–147. 10.1016/S2215-0366(20)30004-3 31974072

[29] Wong AYS , Wong ICK , Chui CSL , Lee EHM , Chang WC , Chen EYH , et al. Association between acute neuropsychiatric events and *helicobacter pylori* therapy containing clarithromycin. JAMA Intern Med. 2016;176(6):828–834. 27136661 10.1001/jamainternmed.2016.1586

[30] The Health Improvement Network . Fostering advancements in research, patient care and outcomes . Leyland: Health Improvement Network; 2023 https://www.the‐health‐improvement‐network.com/.

[31] Lewis JD , Schinnar R , Bilker WB , Wang X , Strom BL . Validation studies of the Health Improvement Network (THIN) database for pharmacoepidemiology research. Pharmacoepidemiol Drug Saf. 2007;16(4):393–401. 17066486 10.1002/pds.1335

[32] Blak BT , Thompson M , Dattani H , Bourke A . Generalisability of The Health Improvement Network (THIN) database: demographics, chronic disease prevalence and mortality rates. Inform Prim Care. 2011;19(4):251–255. 22828580 10.14236/jhi.v19i4.820

[33] Laurent T , Simeone J , Kuwatsuru R , Hirano T , Graham S , Wakabayashi R , et al. Context and considerations for use of two Japanese real‐world databases in Japan: medical data vision and Japanese medical data center. Drugs Real World Outcomes. 2022;9(2):175–187. 35304702 10.1007/s40801-022-00296-5 PMC8932467

[34] Yamana H , Konishi T , Yasunaga H . Validation studies of Japanese administrative health care data: a scoping review. Pharmacoepidemiol Drug Saf. 2023;32(7):705–717. 37146098 10.1002/pds.5636

[35] Iwagami M , Kumazawa R , Miyamoto Y , Ito Y , Ishimaru M , Morita K , et al. Risk of cancer in association with ranitidine and nizatidine vs other H2 blockers: analysis of the Japan medical data center claims database 2005–2018. Drug Saf. 2021;44(3):361–371. 33247391 10.1007/s40264-020-01024-0

[36] Li X , Ostropolets A , Makadia R , Shoaibi A , Rao G , Sena AG , et al. Characterising the background incidence rates of adverse events of special interest for covid‐19 vaccines in eight countries: multinational network cohort study. BMJ. 2021;373:n1435. 35727911 10.1136/bmj.n1435 PMC8193077

[37] Farrington P , Whitaker H , Weldeselassie YG . Self‐controlled case series studies: a modelling guide with R. Boca Raton (FL): CRC Press; 2018.

[38] Wan EYF , Chui CSL , Lai FTT , Chan EWY , Li X , Yan VKC , et al. Bell's palsy following vaccination with mRNA (BNT162b2) and inactivated (CoronaVac) SARS‐CoV‐2 vaccines: a case series and nested case‐control study. Lancet Infect Dis. 2022;22(1):64–72. 34411532 10.1016/S1473-3099(21)00451-5 PMC8367195

[39] Wan EYF , Chui CSL , Wang Y , Ng V WS , Yan VKC , Lai FTT , et al. Herpes zoster related hospitalization after inactivated (CoronaVac) and mRNA (BNT162b2) SARS‐CoV‐2 vaccination: a self‐controlled case series and nested case‐control study. Lancet Reg Health West Pac. 2022;21:100393. 35128500 10.1016/j.lanwpc.2022.100393 PMC8808060

[40] Man KKC , Lau WCY , Coghill D , Besag FMC , Cross JH , Ip P , et al. Association between methylphenidate treatment and risk of seizure: A population‐based, self‐controlled case‐series study. Lancet Child Adolesc Health. 2020;4(6):435–443. 32450123 10.1016/S2352-4642(20)30100-0

[41] Chui CSL , Fan M , Wan EYF , Leung MTY , Cheung E , Yan VKaC , et al. Thromboembolic events and hemorrhagic stroke after mRNA (BNT162b2) and inactivated (CoronaVac) covid‐19 vaccination: a self‐controlled case series study. eClinicalMedicine. 2022;50:101504. 35770253 10.1016/j.eclinm.2022.101504 PMC9233170

[42] Wan EYF , Wang Y , Chui CSL , Mok AHY , Xu W , Yan VKC , et al. Safety of an inactivated, whole‐virion COVID‐19 vaccine (CoronaVac) in people aged 60 years or older in Hong Kong: a modified self‐controlled case series. Lancet Healthy Longev. 2022;3(7):e491–e500. 35813276 10.1016/S2666-7568(22)00125-8 PMC9252509

[43] Grundy SM , Stone NJ , Bailey AL , Beam C , Birtcher KK , Blumenthal RS , et al. 2018 AHA/ACC/AACVPR/AAPA/ABC/ACPM/ADA/AGS/APhA/ASPC/NLA/PCNA guideline on the management of blood cholesterol: executive summary: a report of the American College of Cardiology/American Heart Association task force on Clinical Practice Guidelines. Circulation. 2019;139(25):e1046–e1081. 30565953 10.1161/CIR.0000000000000624

[44] Yu YL , Hawkins BR , Ip MS , Wong V , Woo E . Myasthenia gravis in Hong Kong Chinese. 1. Epidemiology and adult disease. Acta Neurol Scand. 1992;86(2):113–119. 1414218 10.1111/j.1600-0404.1992.tb05050.x

[45] Carey IM , Banchoff E , Nirmalananthan N , Harris T , DeWilde S , Chaudhry UAR , et al. Prevalence and incidence of neuromuscular conditions in the UK between 2000 and 2019: a retrospective study using primary care data. PLoS One. 2021;16(12):e0261983. 34972157 10.1371/journal.pone.0261983 PMC8719665

[46] Matsui N , Nakane S , Nakagawa Y , Kondo K , Mitsui T , Matsumoto T , et al. Increasing incidence of elderly onset patients with myasthenia gravis in a local area of Japan. J Neurol Neurosurg Psychiatry. 2009;80(10):1168–1171. 19762910 10.1136/jnnp.2008.152637

[47] Yoshikawa H , Adachi Y , Nakamura Y , Kuriyama N , Murai H , Nomura Y , et al. Two‐step nationwide epidemiological survey of myasthenia gravis in Japan 2018. PLoS One. 2022;17(9):e0274161. 36129914 10.1371/journal.pone.0274161 PMC9491589

[48] Oh SJ , Dhall R , Young A , Morgan MB , Lu L , Claussen GC . Statins may aggravate myasthenia gravis. Muscle Nerve. 2008;38(3):1101–1107. 18720508 10.1002/mus.21074 PMC2670554

[49] Mammen AL . Statin‐associated autoimmune myopathy. N Engl J Med. 2016;374(7):664–669. 26886523 10.1056/NEJMra1515161

[50] Schneeweiss S . Real‐world evidence of treatment effects: The useful and the misleading. Clin Pharmacol Ther. 2019;106(1):43–44. 30942896 10.1002/cpt.1405

