# Supplementary material for: Myasthenia gravis following the initiation of statin therapy: A multinational self‐controlled case series study
Source: J Intern Med. 2026 Feb 5;299(4):502–14. doi: 10.1111/joim.70072 (PMC12950630; doi:10.1111/joim.70072)
Supplement: Supplementary file 1 — Table S1: Case definitions of myasthenia gravis. Table S2: Subgroup and sensitivity analyses results of each individual site Hong Kong. Table S3: Sensitivity analyses pooled results across all sites. Table S4: Definitions for statin intensity. [file JOIM-299-502-s001.docx]

**Supplementary Appendix**

**Supplemental Table 1. Case definitions**

**Supplemental Table 2. Subgroup and sensitivity analyses –results of each individual site**

**Supplemental Table 3. Sensitivity analyses – pooled results across all sites**

**Supplemental Table 4. Definitions for statin intensity**

**Supplemental Table 1. Case definitions of myasthenia gravis**

| **Study site** | **Coding system** | **Codes** | **Operational definition** |
| --- | --- | --- | --- |
| Hong Kong | ICD-9 code | 358.0* | Myasthenia gravis |
| United Kingdom | Read codes | F380.00 | Myasthenia gravis |
|  |  | F380100 | Juvenile or adult myasthenia gravis |
|  |  | F380z00 | Myasthenia gravis NOS |
| Japan | ICD-10 code | G700* | Myasthenia gravis |

**Supplemental Table 2. Subgroup and sensitivity analyses –results of each individual site**

**Hong Kong**

|  | **No. of events** | **Follow-up (person-years)** | **Crude incidence (per person-year)*** | **Incident rate ratio (95% CI)** |
| --- | --- | --- | --- | --- |
| **Sex** |  |  |  |  |
| *Male* |  |  |  |  |
| 0-179 days after initiation | 113 | 307.5 | 0.368 | 4.652 (3.603-6.006) |
| 180-364 days after initiation | 43 | 297.6 | 0.144 | 1.775 (1.249-2.524) |
| 365-729 days after initiation | 62 | 537.0 | 0.115 | 1.338 (0.972-1.842) |
| Non-exposure period | 380 | 8206.8 | 0.046 | (Ref) |
| *Female* |  |  |  |  |
| 0-179 days after initiation | 115 | 322.2 | 0.357 | 4.654 (3.642-5.948) |
| 180-364 days after initiation | 33 | 304.8 | 0.108 | 1.360 (0.927-1.995) |
| 365-729 days after initiation | 37 | 553.1 | 0.067 | 0.785 (0.539-1.142) |
| Non-exposure period | 454 | 8560.6 | 0.053 | (Ref) |
| **Statin intensity** |  |  |  |  |
| *Low-intensity* |  |  |  |  |
| 0-179 days after initiation | 110 | 336.3 | 0.327 | 4.229 (3.291-5.434) |
| 180-364 days after initiation | 42 | 332.1 | 0.126 | 1.594 (1.123-2.262) |
| 365-729 days after initiation | 68 | 631.1 | 0.108 | 1.297 (0.956-1.760) |
| Non-exposure period | 434 | 7963.3 | 0.055 | (Ref) |
| *Medium-intensity* |  |  |  |  |
| 0-179 days after initiation | 114 | 277.6 | 0.411 | 5.172 (4.006-6.679) |
| 180-364 days after initiation | 33 | 255.8 | 0.129 | 1.559 (1.055-2.303) |
| 365-729 days after initiation | 28 | 434.4 | 0.064 | 0.717 (0.469-1.096) |
| Non-exposure period | 378 | 8316.1 | 0.045 | (Ref) |
| *High-intensity* |  |  |  |  |
| 0-179 days after initiation | 4 | 15.7 | 0.255 | 7.028 (1.850-26.707) |
| 180-364 days after initiation | 1 | 14.4 | 0.069 | 1.843 (0.206-16.498) |
| 365-729 days after initiation | 3 | 24.6 | 0.122 | 2.987 (0.610-14.622) |
| Non-exposure period | 22 | 488.1 | 0.045 | (Ref) |
| **Drug** |  |  |  |  |
| *Simvastatin* |  |  |  |  |
| 0-179 days after initiation | 156 | 441.8 | 0.353 | 4.336 (3.505-5.364) |
| 180-364 days after initiation | 59 | 435.9 | 0.135 | 1.618 (1.202-2.177) |
| 365-729 days after initiation | 81 | 824.6 | 0.098 | 1.119 (0.851-1.471) |
| Non-exposure period | 566 | 10397.5 | 0.054 | (Ref) |
| *Atorvastatin* |  |  |  |  |
| 0-179 days after initiation | 60 | 164.1 | 0.366 | 5.597 (3.952-7.926) |
| 180-364 days after initiation | 13 | 143.0 | 0.091 | 1.341 (0.735-2.447) |
| 365-729 days after initiation | 17 | 223.2 | 0.076 | 1.045 (0.600-1.818) |
| Non-exposure period | 240 | 5718.2 | 0.042 | (Ref) |
| *Rosuvastatin* |  |  |  |  |
| 0-179 days after initiation | 11 | 22.3 | 0.494 | 6.425 (2.661-15.514) |
| 180-364 days after initiation | 4 | 21.9 | 0.183 | 2.221 (0.666-7.408) |
| 365-729 days after initiation | 0 | 39.3 | 0.000 | - |
| Non-exposure period | 28 | 651.0 | 0.043 | (Ref) |
| *Fluvastatin* |  |  |  |  |
| 0-179 days after initiation | 1 | 1.5 | 0.676 | - |
| 180-364 days after initiation | 0 | 1.5 | 0.000 | - |
| 365-729 days after initiation | 1 | 3.0 | 0.333 | - |
| Non-exposure period | 0 | 0.6 | 0.000 | (Ref) |
| **Sensitivity analyses** | |  |  |  |
| *Censor on statin discontinuation* | |  |  |  |
| 0-179 days after initiation | 219 | 576.7 | 0.380 | 4.849 (4.052-5.804) |
| 180-364 days after initiation | 58 | 499.3 | 0.116 | 1.481 (1.110-1.977) |
| 365-729 days after initiation | 80 | 839.7 | 0.095 | 1.154 (0.888-1.500) |
| Non-exposure period | 834 | 16312.6 | 0.051 | (Ref) |
| *Exclude death cases* |  |  |  |  |
| 0-179 days after initiation | 216 | 612.75 | 0.353 | 4.589 (3.828-5.500) |
| 180-364 days after initiation | 72 | 592.48 | 0.122 | 1.526 (1.171-1.989) |
| 365-729 days after initiation | 95 | 1081.96 | 0.088 | 1.038 (0.812-1.326) |
| Non-exposure period | 804 | 16169.88 | 0.050 | (Ref) |
| *Day 0 as a separate risk period* | | | | |
| Day of statin initiation | 35 | 3.6 | 9.699 | 125.352 (88.218-178.116) |
| 1-179 days after initiation | 193 | 626.0 | 0.308 | 3.958 (3.289-4.763) |
| 180-364 days after initiation | 76 | 602.3 | 0.126 | 1.566 (1.210-2.028) |
| 365-729 days after initiation | 99 | 1090.1 | 0.091 | 1.060 (0.834-1.349) |
| Non-exposure period | 834 | 16767.5 | 0.050 | (Ref) |
| *Exclude MG events on the same day of statin initiation* | | |  |  |
| 0-179 days after initiation | 193 | 612.7 | 0.315 | 3.929 (3.264-4.730) |
| 180-364 days after initiation | 76 | 585.5 | 0.130 | 1.565 (1.209-2.026) |
| 365-729 days after initiation | 99 | 1058.0 | 0.094 | 1.060 (0.833-1.349) |
| Non-exposure period | 834 | 16371.5 | 0.051 | (Ref) |
| *Pre-risk period of 90 days* |  |  |  |  |
| 0-179 days after initiation | 228 | 629.6 | 0.362 | 4.614 (3.879-5.489) |
| 180-364 days after initiation | 76 | 602.3 | 0.126 | 1.555 (1.204-2.008) |
| 365-729 days after initiation | 99 | 1090.1 | 0.091 | 1.052 (0.829-1.334) |
| Non-exposure period | 862 | 17089.3 | 0.050 | (Ref) |
| *Pre-risk period of 365 days* |  |  |  |  |
| 0-179 days after initiation | 228 | 629.6 | 0.362 | 4.819 (4.009-5.791) |
| 180-364 days after initiation | 76 | 602.3 | 0.126 | 1.626 (1.250-2.117) |
| 365-729 days after initiation | 99 | 1090.1 | 0.091 | 1.104 (0.862-1.414) |
| Non-exposure period | 777 | 16107.6 | 0.048 | (Ref) |
| *MG defined with ≥2 diagnosis codes at different time points* | | | |  |
| 0-179 days after initiation | 144 | 407.3621 | 0.353494 | 4.770 (3.818- 5.959) |
| 180-364 days after initiation | 45 | 394.8583 | 0.113965 | 1.504 (1.078- 2.098) |
| 365-729 days after initiation | 54 | 721.3525 | 0.074859 | 0.943 (0.686- 1.296) |
| Non-exposure period | 554 | 10317.47 | 0.053695 | (Ref) |

***** The reported crude incidence rates should only be interpreted as relative to incidence rates in other risk periods or the non-exposure period, and do not represent the absolute incidence rate in the general population, since this is a case-only study without information on statin non-users or people without MG outcome

**United Kingdom**

|  | **No. of events** | **Follow-up (person-years)** | **Crude incidence (per person-year)*** | **Incident rate ratio (95% CI)** |
| --- | --- | --- | --- | --- |
| **Sex** |  |  |  |  |
| *Male* |  |  |  |  |
| 0-179 days after initiation | 47 | 135.1 | 0.348 | 2.968 (2.009-4.383) |
| 180-364 days after initiation | 30 | 131.0 | 0.229 | 1.887 (1.197-2.974) |
| 365-729 days after initiation | 31 | 233.3 | 0.133 | 1.015 (0.632-1.628) |
| Non-exposure period | 141 | 2179.0 | 0.065 | (Ref) |
| *Female* |  |  |  |  |
| 0-179 days after initiation | 26 | 76.0 | 0.342 | 2.965 (1.754-5.012) |
| 180-364 days after initiation | 12 | 74.4 | 0.161 | 1.407 (0.716-2.762) |
| 365-729 days after initiation | 15 | 137.1 | 0.109 | 0.927 (0.484-1.779) |
| Non-exposure period | 87 | 1327.2 | 0.066 | (Ref) |
| **Statin intensity** |  |  |  |  |
| *Low-intensity* |  |  |  |  |
| 0-179 days after initiation | 6 | 23.9 | 0.251 | 1.708 (0.589-4.956) |
| 180-364 days after initiation | 7 | 23.5 | 0.298 | 1.966 (0.694-5.570) |
| 365-729 days after initiation | 4 | 42.5 | 0.094 | 0.506 (0.135-1.902) |
| Non-exposure period | 23 | 261.0 | 0.088 | (Ref) |
| *Medium-intensity* |  |  |  |  |
| 0-179 days after initiation | 59 | 167.3 | 0.353 | 3.140 (2.215-4.452) |
| 180-364 days after initiation | 33 | 163.1 | 0.202 | 1.781 (1.167-2.716) |
| 365-729 days after initiation | 42 | 294.6 | 0.143 | 1.194 (0.792-1.799) |
| Non-exposure period | 181 | 2753.8 | 0.066 | (Ref) |
| *High-intensity* |  |  |  |  |
| 0-179 days after initiation | 7 | 19.4 | 0.360 | 3.918 (1.450-10.584) |
| 180-364 days after initiation | 2 | 18.4 | 0.109 | 1.055 (0.220-5.061) |
| 365-729 days after initiation | 0 | 32.3 | 0.000 | - |
| Non-exposure period | 24 | 491.4 | 0.049 | (Ref) |
| **Drug** |  |  |  |  |
| *Simvastatin* |  |  |  |  |
| 0-179 days after initiation | 48 | 135.5 | 0.354 | 3.431 (2.308-5.101) |
| 180-364 days after initiation | 27 | 134.4 | 0.201 | 1.931 (1.198-3.111) |
| 365-729 days after initiation | 32 | 247.4 | 0.129 | 1.183 (0.734-1.907) |
| Non-exposure period | 141 | 1987.6 | 0.071 | (Ref) |
| *Atorvastatin* |  |  |  |  |
| 0-179 days after initiation | 21 | 63.8 | 0.329 | 2.672 (1.526-4.679) |
| 180-364 days after initiation | 13 | 59.3 | 0.219 | 1.677 (0.862-3.262) |
| 365-729 days after initiation | 11 | 101.7 | 0.108 | 0.765 (0.367-1.593) |
| Non-exposure period | 76 | 1407.0 | 0.054 | (Ref) |
| *Rosuvastatin* |  |  |  |  |
| 0-179 days after initiation | 2 | 3.9 | 0.507 | 8.925 (0.531-149.920) |
| 180-364 days after initiation | 0 | 4.1 | 0.000 | - |
| 365-729 days after initiation | 2 | 8.0 | 0.249 | 4.449 (0.218-90.709) |
| Non-exposure period | 3 | 38.9 | 0.077 | (Ref) |
| *Pravastatin* |  |  |  |  |
| 0-179 days after initiation | 1 | 5.9 | 0.169 | 0.673 (0.066-6.826) |
| 180-364 days after initiation | 0 | 5.9 | 0.000 | - |
| 365-729 days after initiation | 1 | 10.3 | 0.097 | 0.406 (0.029-5.643) |
| Non-exposure period | 8 | 69.2 | 0.116 | (Ref) |
| *Fluvastatin* |  |  |  |  |
| 0-179 days after initiation | 1 | 1.5 | 0.676 | - |
| 180-364 days after initiation | 1 | 1.2 | 0.853 | - |
| 365-729 days after initiation | 0 | 2.0 | 0.000 | - |
| Non-exposure period | 0 | 3.2 | 0.000 | (Ref) |
| *Cerivastatin* |  |  |  |  |
| 0-179 days after initiation | 0 | 0.5 | 0.000 | - |
| 180-364 days after initiation | 1 | 0.5 | 1.974 | - |
| 365-729 days after initiation | 0 | 1.0 | 0.000 | - |
| Non-exposure period | 0 | 0.4 | 0.000 | (Ref) |
| **Sensitivity analyses** | |  |  |  |
| *Censor on statin discontinuation* | |  |  |  |
| 0-179 days after initiation | 64 | 176.3 | 0.363 | 2.850 (2.053-3.957) |
| 180-364 days after initiation | 30 | 142.6 | 0.210 | 1.744 (1.134-2.680) |
| 365-729 days after initiation | 28 | 227.5 | 0.123 | 0.987 (0.624-1.561) |
| Non-exposure period | 228 | 3212.8 | 0.071 | (Ref) |
| *Exclude death cases* |  |  |  |  |
| 0-179 days after initiation | 70 | 196.59 | 0.356 | 3.149 (2.287-4.337) |
| 180-364 days after initiation | 40 | 193.69 | 0.207 | 1.774 (1.206-2.608) |
| 365-729 days after initiation | 46 | 364.49 | 0.126 | 1.016 (0.691-1.495) |
| Non-exposure period | 210 | 3309.10 | 0.063 | (Ref) |
| *Day 0 as a separate risk period* | | | | |
| Day of statin initiation | 5 | 1.2 | 4.218 | 37.218 (15.124-91.592) |
| 1-179 days after initiation | 68 | 209.9 | 0.324 | 2.827 (2.056-3.887) |
| 180-364 days after initiation | 42 | 205.4 | 0.204 | 1.756 (1.207-2.555) |
| 365-729 days after initiation | 46 | 370.4 | 0.124 | 1.011 (0.691-1.479) |
| Non-exposure period | 228 | 3506.2 | 0.065 | (Ref) |
| *Exclude MG events on the same day of statin initiation* | | |  |  |
| 0-179 days after initiation | 68 | 208.6 | 0.326 | 2.839 (2.065-3.905) |
| 180-364 days after initiation | 42 | 202.9 | 0.207 | 1.776 (1.220-2.584) |
| 365-729 days after initiation | 46 | 365.4 | 0.126 | 1.024 (0.700-1.499) |
| Non-exposure period | 228 | 3490.3 | 0.065 | (Ref) |
| *Pre-risk period of 90 days* |  |  |  |  |
| 0-179 days after initiation | 73 | 211.1 | 0.346 | 2.843 (2.099-3.851) |
| 180-364 days after initiation | 42 | 205.4 | 0.204 | 1.650 (1.143-2.382) |
| 365-729 days after initiation | 46 | 370.4 | 0.124 | 0.945 (0.651-1.370) |
| Non-exposure period | 246 | 3611.2 | 0.068 | (Ref) |
| *Pre-risk period of 365 days* |  |  |  |  |
| 0-179 days after initiation | 73 | 211.1 | 0.346 | 2.803 (2.023-3.883) |
| 180-364 days after initiation | 42 | 205.4 | 0.204 | 1.627 (1.104-2.397) |
| 365-729 days after initiation | 46 | 370.4 | 0.124 | 0.930 (0.626-1.381) |
| Non-exposure period | 211 | 3292.4 | 0.064 | (Ref) |
| *MG defined with ≥2 diagnosis codes at different time points* | | | |  |
| 0-179 days after initiation | 36 | 109.3443 | 0.329235 | 2.691 (1.751-4.136) |
| 180-364 days after initiation | 19 | 107.4716 | 0.176791 | 1.417 (0.828-2.427) |
| 365-729 days after initiation | 19 | 188.9117 | 0.100576 | 0.767 (0.437-1.346) |
| Non-exposure period | 126 | 1676.898 | 0.075139 | (Ref) |

***** The reported crude incidence rates should only be interpreted as relative to incidence rates in other risk periods or the non-exposure period, and do not represent the absolute incidence rate in the general population, since this is a case-only study without information on statin non-users or people without MG outcome

**Japan**

|  | **No. of events** | **Follow-up (person-years)** | **Crude incidence (per person-year)*** | **Incident rate ratio (95% CI)** |
| --- | --- | --- | --- | --- |
| **Sex** |  |  |  |  |
| *Male* |  |  |  |  |
| 0-179 days after initiation | 49 | 138.3 | 0.354 | 1.243 (0.805-1.920) |
| 180-364 days after initiation | 39 | 130.4 | 0.299 | 1.050 (0.656-1.679) |
| 365-729 days after initiation | 50 | 216.5 | 0.231 | 0.773 (0.478-1.251) |
| Non-exposure period | 105 | 946.6 | 0.111 | (Ref) |
| *Female* |  |  |  |  |
| 0-179 days after initiation | 40 | 106.9 | 0.374 | 1.332 (0.821-2.161) |
| 180-364 days after initiation | 23 | 100.0 | 0.230 | 0.866 (0.492-1.525) |
| 365-729 days after initiation | 44 | 159.7 | 0.276 | 1.047 (0.621-1.766) |
| Non-exposure period | 95 | 751.5 | 0.126 | (Ref) |
| **Statin intensity** |  |  |  |  |
| *Low-intensity* |  |  |  |  |
| 0-179 days after initiation | 55 | 148.6 | 0.370 | 1.262 (0.840-1.896) |
| 180-364 days after initiation | 38 | 138.6 | 0.274 | 0.949 (0.601-1.498) |
| 365-729 days after initiation | 52 | 221.7 | 0.235 | 0.795 (0.504-1.254) |
| Non-exposure period | 125 | 1001.2 | 0.125 | (Ref) |
| *Medium-intensity* |  |  |  |  |
| 0-179 days after initiation | 34 | 96.6 | 0.352 | 1.430 (0.843-2.428) |
| 180-364 days after initiation | 24 | 91.8 | 0.261 | 1.093 (0.609-1.963) |
| 365-729 days after initiation | 42 | 154.6 | 0.272 | 1.108 (0.632-1.943) |
| Non-exposure period | 75 | 696.9 | 0.108 | (Ref) |
| **Drug** |  |  |  |  |
| *Simvastatin* |  |  |  |  |
| 0-179 days after initiation | 0 | 3.4 | 0.000 | - |
| 180-364 days after initiation | 1 | 3.5 | 0.282 | 0.631 (0.037-10.823) |
| 365-729 days after initiation | 2 | 6.1 | 0.327 | 0.552 (0.035-8.703) |
| Non-exposure period | 3 | 13.8 | 0.217 | (Ref) |
| *Atorvastatin* |  |  |  |  |
| 0-179 days after initiation | 24 | 67.1 | 0.358 | 0.929 (0.492-1.754) |
| 180-364 days after initiation | 18 | 63.4 | 0.284 | 0.774 (0.387-1.547) |
| 365-729 days after initiation | 30 | 102.9 | 0.291 | 0.818 (0.417-1.607) |
| Non-exposure period | 53 | 395.6 | 0.134 | (Ref) |
| *Rosuvastatin* |  |  |  |  |
| 0-179 days after initiation | 38 | 107.3 | 0.354 | 1.339 (0.827-2.166) |
| 180-364 days after initiation | 24 | 98.5 | 0.244 | 0.916 (0.526-1.595) |
| 365-729 days after initiation | 37 | 158.2 | 0.234 | 0.823 (0.480-1.413) |
| Non-exposure period | 92 | 796.8 | 0.115 | (Ref) |
| *Pravastatin* |  |  |  |  |
| 0-179 days after initiation | 10 | 22.4 | 0.446 | 0.978 (0.321-2.980) |
| 180-364 days after initiation | 6 | 21.3 | 0.281 | 0.650 (0.187-2.260) |
| 365-729 days after initiation | 7 | 32.7 | 0.214 | 0.493 (0.140-1.732) |
| Non-exposure period | 19 | 139.3 | 0.136 | (Ref) |
| *Pitavastatin* |  |  |  |  |
| 0-179 days after initiation | 17 | 44.4 | 0.383 | 2.293 (1.077-4.882) |
| 180-364 days after initiation | 13 | 43.2 | 0.301 | 1.839 (0.807-4.189) |
| 365-729 days after initiation | 17 | 75.3 | 0.226 | 1.345 (0.577-3.135) |
| Non-exposure period | 33 | 349.4 | 0.094 | (Ref) |
| *Fluvastatin* |  |  |  |  |
| 0-179 days after initiation | 0 | 0.5 | 0.000 | - |
| 180-364 days after initiation | 0 | 0.5 | 0.000 | - |
| 365-729 days after initiation | 0 | 0.5 | 0.000 | - |
| Non-exposure period | 1 | 1.0 | 0.998 | (Ref) |
| **Sensitivity analyses** | |  |  |  |
| *Censor on statin discontinuation* | |  |  |  |
| 0-179 days after initiation | 88 | 228.0 | 0.386 | 1.257 (0.904-1.748) |
| 180-364 days after initiation | 52 | 190.7 | 0.273 | 0.929 (0.632-1.365) |
| 365-729 days after initiation | 75 | 281.8 | 0.266 | 0.894 (0.610-1.310) |
| Non-exposure period | 200 | 1623.9 | 0.123 | (Ref) |
| *Exclude death cases* |  |  |  |  |
| 0-179 days after initiation | 88 | 244.21 | 0.360 | 1.275 (0.923-1.762) |
| 180-364 days after initiation | 62 | 229.86 | 0.270 | 0.979 (0.683-1.402) |
| 365-729 days after initiation | 94 | 376.24 | 0.250 | 0.889 (0.625-1.265) |
| Non-exposure period | 200 | 1694.83 | 0.118 | (Ref) |
| *Day 0 as a separate risk period* | | | | |
| Day of statin initiation | 8 | 1.4 | 5.663 | 20.205 (9.700-42.085) |
| 1-179 days after initiation | 81 | 243.8 | 0.332 | 1.188 (0.855-1.650) |
| 180-364 days after initiation | 62 | 230.4 | 0.269 | 0.986 (0.688-1.411) |
| 365-729 days after initiation | 94 | 376.2 | 0.250 | 0.898 (0.631-1.277) |
| Non-exposure period | 200 | 1698.1 | 0.118 | (Ref) |
| *Exclude MG events on the same day of statin initiation* | | |  |  |
| 0-179 days after initiation | 81 | 241.3 | 0.336 | 1.184 (0.851-1.647) |
| 180-364 days after initiation | 62 | 226.4 | 0.274 | 0.996 (0.695-1.429) |
| 365-729 days after initiation | 94 | 369.0 | 0.255 | 0.911 (0.639-1.298) |
| Non-exposure period | 200 | 1687.7 | 0.119 | (Ref) |
| *Pre-risk period of 90 days* |  |  |  |  |
| 0-179 days after initiation | 89 | 245.2 | 0.363 | 1.218 (0.894-1.659) |
| 180-364 days after initiation | 62 | 230.4 | 0.269 | 0.923 (0.653-1.306) |
| 365-729 days after initiation | 94 | 376.2 | 0.250 | 0.837 (0.597-1.173) |
| Non-exposure period | 226 | 1791.5 | 0.126 | (Ref) |
| *Pre-risk period of 365 days* |  |  |  |  |
| 0-179 days after initiation | 89 | 245.2 | 0.363 | 1.529 (1.076-2.172) |
| 180-364 days after initiation | 62 | 230.4 | 0.269 | 1.170 (0.794-1.725) |
| 365-729 days after initiation | 94 | 376.2 | 0.250 | 1.081 (0.735-1.589) |
| Non-exposure period | 155 | 1515.4 | 0.102 | (Ref) |
| *MG defined with ≥2 diagnosis codes at different time points* | | | |  |
| 0-179 days after initiation | 70 | 186.6338 | 0.375066 | 1.202 (0.837-1.726) |
| 180-364 days after initiation | 54 | 174.5982 | 0.309282 | 0.978 (0.659-1.453) |
| 365-729 days after initiation | 72 | 283.1129 | 0.254315 | 0.737 (0.491-1.107) |
| Non-exposure period | 143 | 1198.93 | 0.119273 |  |

***** The reported crude incidence rates should only be interpreted as relative to incidence rates in other risk periods or the non-exposure period, and do not represent the absolute incidence rate in the general population, since this is a case-only study without information on statin non-users or people without MG outcome

**Supplemental Table 3. Sensitivity analyses – pooled results across all sites**

|  | **No. of events** | **Follow-up (person-years)** | **Crude incidence (per person-year)*** | **Incident rate ratio (95% CI)** |
| --- | --- | --- | --- | --- |
| *Censor on statin discontinuation* | |  |  |  |
| 0-179 days after initiation | 371 | 981.0 | 0.378 | 2.613 (1.207-5.658) |
| 180-364 days after initiation | 140 | 832.6 | 0.168 | 1.338 (0.941-1.901) |
| 365-729 days after initiation | 183 | 1349.0 | 0.136 | 1.049 (0.863-1.276) |
| Non-exposure period | 1262 | 21149.2 | 0.060 | (Ref) |
| *Exclude death cases* |  |  |  |  |
| 0-179 days after initiation | 374 | 1053.6 | 0.355 | 2.663 (1.264-5.610) |
| 180-364 days after initiation | 174 | 1016.0 | 0.171 | 1.387 (0.995-1.933) |
| 365-729 days after initiation | 235 | 1822.7 | 0.129 | 0.993 (0.831-1.187) |
| Non-exposure period | 1214 | 21173.8 | 0.057 | (Ref) |
| *Day 0 as a separate risk period* | | | | |
| Day of statin initiation | 48 | 6.2 | 7.734 | 47.651 (15.786-143.836) |
| 1-179 days after initiation | 342 | 1079.7 | 0.317 | 2.390 (1.185-4.823) |
| 180-364 days after initiation | 180 | 1038.2 | 0.173 | 1.402 (1.009-1.948) |
| 365-729 days after initiation | 239 | 1836.7 | 0.130 | 1.007 (0.844-1.201) |
| Non-exposure period | 1262 | 21971.8 | 0.057 | (Ref) |
| *Exclude MG events on the same day of statin initiation* | | |  |  |
| 0-179 days after initiation | 342 | 1062.6 | 0.322 | 2.385 (1.183-4.808) |
| 180-364 days after initiation | 180 | 1014.7 | 0.177 | 1.412 (1.019-1.955) |
| 365-729 days after initiation | 239 | 1792.4 | 0.133 | 1.013 (0.849-1.209) |
| Non-exposure period | 1262 | 21549.6 | 0.059 | (Ref) |
| *Pre-risk period of 90 days* |  |  |  |  |
| 0-179 days after initiation | 390 | 1086.0 | 0.359 | 2.538 (1.182-5.451) |
| 180-364 days after initiation | 180 | 1038.2 | 0.173 | 1.340 (0.943-1.905) |
| 365-729 days after initiation | 239 | 1836.7 | 0.130 | 0.969 (0.815-1.151) |
| Non-exposure period | 1334 | 22492.0 | 0.059 | (Ref) |
| *Pre-risk period of 365 days* |  |  |  |  |
| 0-179 days after initiation | 390 | 1086.0 | 0.359 | 2.781 (1.446-5.346) |
| 180-364 days after initiation | 180 | 1038.2 | 0.173 | 1.503 (1.243-1.817) |
| 365-729 days after initiation | 239 | 1836.7 | 0.130 | 1.059 (0.880-1.273) |
| Non-exposure period | 1143 | 20915.4 | 0.055 | (Ref) |
| *MG defined with ≥2 diagnosis codes at different time points* | | | |  |
| 0-179 days after initiation | 250 | 703.3402 | 0.355447 | 2.512 (1.135-5.559) |
| 180-364 days after initiation | 118 | 676.9281 | 0.174317 | 1.278 (0.957-1.706) |
| 365-729 days after initiation | 145 | 1193.377 | 0.121504 | 0.843 (0.670-1.060) |
| Non-exposure period | 823 | 13193.3 | 0.06238 | (Ref) |

***** The reported crude incidence rates should only be interpreted as relative to incidence rates in other risk periods or the non-exposure period, and do not represent the absolute incidence rate in the general population, since this is a case-only study without information on statin non-users or people without MG outcome

**Supplemental Table 4. Definitions for statin intensity**

Statin intensity used in this study was defined according to the 2018 American College of Cardiology/American Heart Association (ACC/AHA) Guideline on the Management of Blood Cholesterol, where it is classified based on the expected percentage reduction in low-density lipoprotein cholesterol (LDL-C):

- High-intensity statin therapy: expected to lower LDL-C by ≥50%
- Moderate-intensity statin therapy: expected to lower LDL-C by 30% to 49%
- Low-intensity statin therapy: expected to lower LDL-C by <30%

The specific statins and corresponding doses for each intensity is shown in the following table:

|  | **High Intensity** | **Medium Intensity** | **Low Intensity** |
| --- | --- | --- | --- |
| **LDL-C lowering** | ≥50% | 30%–49% | <30% |
| **Statins** | Atorvastatin (40 mg) 80 mg  Rosuvastatin 20 mg (40 mg) | Atorvastatin 10 mg (20 mg)  Rosuvastatin (5 mg) 10 mg  Simvastatin 20–40 mg | Simvastatin 10 mg |
|  | … | Pravastatin 40 mg (80 mg)  Lovastatin 40 mg (80 mg)  Fluvastatin XL 80 mg  Fluvastatin 40 mg BID  Pitavastatin 1–4 mg | Pravastatin 10–20 mg  Lovastatin 20 mg  Fluvastatin 20–40 mg |

**References**

Grundy SM, Stone NJ, Bailey AL, et al. 2018 AHA/ACC/AACVPR/AAPA/ABC/ACPM/ADA/AGS/APhA/ASPC/NLA/PCNA Guideline on the Management of Blood Cholesterol: A Report of the American College of Cardiology/American Heart Association Task Force on Clinical Practice Guidelines. Circulation. 2019;139(25):e1082-e1143
